# Development and validation of the first needs-based quality of life measure for individuals with manifest Huntington’s disease

**DOI:** 10.1007/s00415-026-13898-8

**Published:** 2026-06-11

**Authors:** Isobel Spray, Mariusz T. Grzeda, Jeanette Thorpe, Ellie Johnstone, Wiebke Hannemann, Ferdinando Squitieri, Giulia Giancaterino, Paola Emilia Mina, Barbara D’Alessio, Astri Arnesen, Jiří Klempíř, Olga Ulmanová, Pearl van Lonkhuizen, Anne-Wil Heemskerk, Jennifer Hoblyn, Emer O’Malley, Ramona Moldovan, Georg Bernhard Landwehrmeyer, Stephen P. McKenna, Alzbeta Mühlbäck

**Affiliations:** 1 Galen Research, Manchester, UK; 2 Department of Neurology, University Hospital Ulm, University Ulm, Ulm, Germany; 3 Unità Huntington E Malattie Rare Fondazione IRCCS Casa Sollievo Della Sofferenza Hospital, San Giovanni Rotondo, Italy; 4 Qrare Medical Centre, Fondazione Lega Italiana Ricerca Huntington (LIRH), Rome, Italy; 5 European Huntington Association, Kristiansand, Norway; 6 Department of Neurology and Centre of Clinical Neuroscience, General University Hospital and First Faculty of Medicine, Charles University, Prague, Czech Republic; 7 Department of Public Health and Primary Care, Leiden University Medical Center, Leiden, Netherlands; 8 National eHealth Living Lab, Leiden University Medical Center, Leiden, Netherlands; 9 Huntington Center Topaz Overduin, Katwijk, Netherlands; 10 Trinity College Dublin, Dublin, Ireland; 11 John of God University Hospital, Dublin, Ireland; 12 Division of Evolution, Infection and Genomics, School of Biological Sciences, University of Manchester, Manchester, UK; 13 Manchester Centre for Genomic Medicine, St. Mary’s Hospital, Manchester University NHS Foundation Trust, Manchester, UK; 14 Department of Psychology, Babeș-Bolyai University, Cluj-Napoca-Napoca, Romania; 15 Huntington-Zentrum Süd, kbo Isar-Amper-Klinikum, Taufkirchen, Germany

**Keywords:** Huntington’s disease, Patient-reported outcome measures (PROM), Quality of life (QoL), Needs-based QoL, Patient value

## Abstract

**Background:**

Huntington’s disease (HD) is a progressive, hereditary neurodegenerative disorder currently without curative treatments, thus making quality of life (QoL) an important outcome for clinical care and therapeutic evaluation. Existing HD-specific patient-reported outcome measures (PROMs) do not adequately capture the experiences of individuals with manifest HD. This study presents the multinational development and validation of the Huntington’s Disease Manifest Quality of Life measure (HD-mQoL), the first needs-based, disease-specific PROM for assessing QoL in individuals with manifest HD.

**Methods:**

Development followed three stages: (1) generation of measure content and translations; (2) testing of face and content validity; and (3) psychometric validation using data from a large international survey analysed with Rasch Measurement Theory (RMT) and Classical Test Theory (CTT).

**Results:**

The measure was completed by 238 individuals with manifest HD from the Czech Republic, Germany, Ireland, Italy, and the UK (59% male; age range 20–83 years). Rasch analysis reduced 49 items to a final set of 23, demonstrating good model fit (item–trait interaction χ^2^ = 0.391), unidimensionality, no differential item functioning, no local dependency, and excellent reliability (Cronbach’s α = 0.91 at timepoint 1, 0.92 at timepoint 2; test–retest r = 0.87). The final measure showed minimal floor and ceiling effects and correlated moderately to strongly with relevant Nottingham Health Profile domains. HD-mQoL scores effectively differentiated subgroups by self-rated disease severity and general health (p < 0.001).

**Conclusions:**

The HD-mQoL is a robust, needs-based measure of QoL, suitable for international use in clinical practice and trials assessing treatment value from the patient’s perspective.

## Introduction

Huntington’s disease (HD) is a rare, progressive, autosomal dominant neurodegenerative disorder that affects an estimated 10 individuals per 100,000 in Western populations [1]. It typically manifests in adulthood and is characterised by a triad of motor, cognitive, and psychiatric symptoms [2]. Once manifest, the disease progresses inexorably, leading to increasing dependence on caregivers and premature death, typically occurring two decades after the onset of disease-specific motor symptoms [2]. The personal, social, and economic consequences of HD are profound [3, 4], driven by the progressive loss of independence and increasing societal isolation experienced by patients and their families [5]. Currently, several disease-modifying strategies are being investigated, with recent findings from studies of AMT-130 (uniQure) suggesting that gene-silencing therapy may slow disease progression in HD. However, such approaches are not yet widely available to patients [6, 7]. Consequently, multidisciplinary management continues to represent the cornerstone of HD care, addressing the complex motor, cognitive, and psychiatric manifestations that define the disease [8]. In this context, suitable outcome measures are essential for disease assessment, management, and treatment evaluation.

Patient-reported outcome measures (PROMs) provide a structured method for capturing the patient’s perspective on disease burden and treatment outcomes and are increasingly incorporated into neurological research and clinical trials [9]. The importance of these measures is highlighted by the U.S. Food and Drug Administration (FDA) guidance, which emphasises the need to advance the collection of robust and meaningful patient input to inform medical product development and regulatory decision-making [10]. Symptom- and function-based PROMs are valuable for evaluating disease or health status, referring to an individual’s functioning across physical, mental, and social domains, and can be reported either by the individual or by proxy perspective [9]. However, evidence suggests that such measures may not fully capture the lived experience of individuals with HD [11]. Anosognosia, a lack of awareness of deficits, has been shown to affect patients’ insight into certain symptoms [11, 12]. In addition, research shows that ratings of symptom importance in HD vary from patient to patient [13]. Consequently, relying solely upon symptom and functioning measures to assess patient value of treatments risks making inaccurate assumptions about HD patients’ experience of their disease [14].

Quality of life (QoL), in contrast, extends beyond the description of functional abilities to encompass an individual’s subjective perception and evaluation of their well-being [15]. QoL assessment, therefore, can capture a more holistic understanding of how HD impacts everyday life. It is well-documented that HD can have a significant impact on QoL [4] with findings suggesting that QoL is exceptionally poor for HD patients, when compared to patients with other neurodegenerative conditions [16].

To date, three HD-specific, validated measures of health-related quality of life (HRQoL) have been developed. The first disease-specific patient-reported HRQoL instrument, the Huntington’s Disease Health-related Quality of Life questionnaire (HDQoL), was developed and validated by Hocaoglu, Gaffan, and Ho (2012b), with a corresponding proxy version published in the same year [17]. Shortly thereafter, the Huntington Quality of Life Instrument (H-QoL-I) was developed and validated, based on findings from the European HD Burden Study [18]. More recently, HDQLIFE was introduced by Carlozzi et al. [19], integrating the validated Neuro-QoL and PROMIS measurement systems with five additional HD-specific scales to provide a comprehensive assessment of HRQoL in this population. These instruments were developed using heterogeneous samples that included individuals at risk for HD, premanifest patients, and those with manifest disease, which included individuals at varying stages of disease progression. However, emerging evidence suggests that further stratification may improve measurement precision, as patient experiences and perceptions of QoL differ across disease stages [4, 20]. Consequently, some items may be less relevant to particular subgroups, potentially limiting the sensitivity and applicability of these tools in specific clinical or research contexts [11]. A further limitation is that these measures are multidimensional and do not yield a single summary score, complicating interpretation and longitudinal measurement of outcomes. This poses challenges for routine clinical practice and clinical trial research settings, for which questionnaire length and ease of score aggregation are important factors [21, 22]. Long or complex tools also burden patients, particularly HD patients with cognitive impairments [11].

There is, therefore, a continuing need to develop additional measures to assess QoL in HD, ensuring that they accurately reflect stage-specific challenges and the lived experiences of affected individuals. QoL is a broader construct that extends beyond medical aspects to encompass social, relational, and occupational facets, including interpersonal relationships and career experiences [23]. This also aligns with the World Health Organization’s (WHO) definition of QoL as “individuals’ perception of their position in life in the context of the culture and value systems in which they live and in relation to their goals, expectations, standards, and concerns” [24].

The needs-based model of QoL, used in the current project, encompasses this definition by using a holistic theoretical framework. Developed in a study of patients diagnosed with depression, the model proposes that QoL is derived from the ability to meet basic human needs [25]. The model has since been successfully applied across several disease areas in the development of disease-specific QoL measures [26–28]. Whilst HRQoL is inherently multidimensional, an advantage of the needs-based model is that it enables the development of a unidimensional measure [29]. Although individual items reflect different areas of need, the overall QoL score represents a person’s ability to fulfil these fundamental needs. A higher score on the measure indicates greater difficulty in meeting needs, and, therefore, poorer QoL. This approach allows for the calculation of a single composite score, which is valuable for monitoring change over time [30]. Needs-based measures have, therefore, been successfully applied in research and clinical trials across a range of disease groups [31–33].

Needs-based measures also offer the unique advantage of assessing patient-perceived value across a variety of intervention types. This is particularly important for outcome assessment in HD, as symptoms, rate of progression, and age of onset can vary widely between individuals [2]. Consequently, treatment approaches are highly heterogeneous, encompassing pharmacological therapies as well as non-pharmacological interventions, such as physiotherapy, occupational therapy, and speech therapy. In this context, healthcare professionals emphasise the importance of keeping the patient’s voice central in the design and delivery of multidisciplinary care [8].

This paper outlines the development and validation of the first needs-based QoL measure for individuals with manifest Huntington’s disease (HD-mQoL), utilising Rasch modelling to examine scaling characteristics and classical test theory to evaluate traditional psychometric properties of the measure.

## Methods

### Study population

Participants were recruited as part of the multinational HEALTHE–RND project, conducted within the framework of the EU Joint Programme–Neurodegenerative Disease (JPND) research initiative. Recruitment took place across six participating countries: the Czech Republic, Germany, Italy, Ireland, the Netherlands, and the United Kingdom.

Eligible participants were adults aged 18 years or older who demonstrated adequate comprehension of the primary language spoken in their country (Czech, Dutch, English, German, or Italian) and were able to provide informed consent. Inclusion criteria required a confirmed genetic diagnosis of HD, defined by the presence of a cytosine–adenine–guanine (CAG) repeat expansion of ≥ 36 in the Huntingtin (*HTT*) gene, together with clinical motor features consistent with manifest HD, as indicated by a Diagnostic Confidence Level (DCL) score of 4 on the Unified Huntington’s Disease Rating Scale (UHDRS)[34].

Exclusion criteria included the presence of any serious psychiatric, neurological, sensory, or metabolic disorder, or any other comorbid condition that could influence QoL. Individuals residing in specialised care facilities were also excluded. The same eligibility criteria were applied consistently across all stages of the study. Participants were identified and recruited through local HD centres in each participating country, with recruitment coordinated by local research teams in accordance with standardised procedures. Ethical approval for the study was obtained from the Research Ethics Committee (REC) for London—West London and GTAC REC in the United Kingdom, and from the respective ethics committees in each participating European country.

### Study stages

**Fig. 1:**
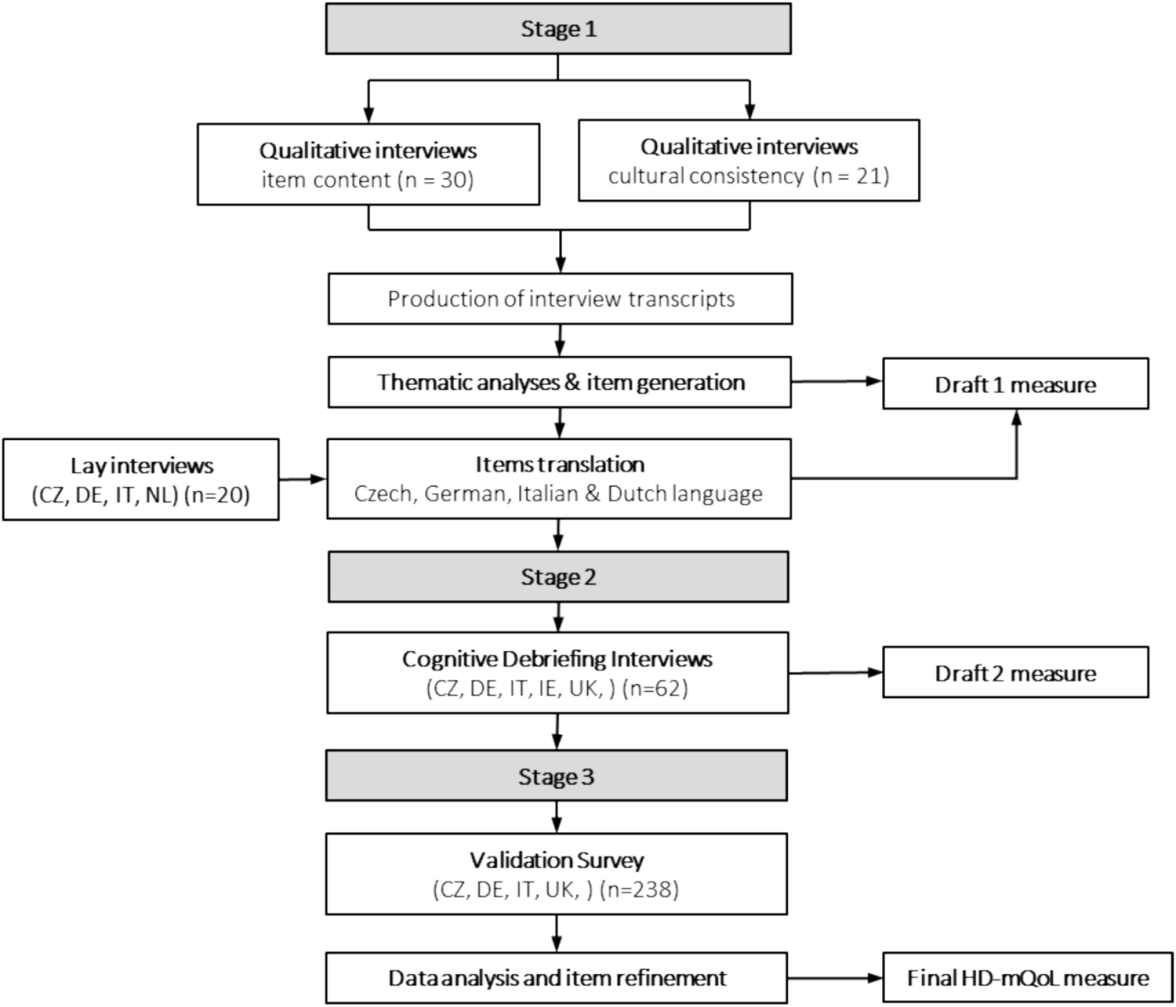
Stages in the Development and Validation of the Huntington’s Disease Manifest Quality of Life (HD-mQoL) measure. CZ (Czech Republic), DE (Germany), IT (Italy), IE (Ireland), NL (Netherlands), UK (United Kingdom), n (number)

The development and validation of the HD-mQoL measure were conducted in three sequential stages. The first stage (1) involved item content generation, aimed at identifying and developing a comprehensive pool of items reflecting the lived experience of individuals with manifest HD. The second stage (2) focused on assessing face and content validity to ensure that items were conceptually appropriate, clearly worded, and representative of the intended construct. In the third stage (3), a draft version of the measure was administered to evaluate its psychometric properties, enabling refinement of the measure and confirmation of the final set of items. An overview of these stages is provided in Fig. 1**.**

#### Stage 1: Item content generation

Item content was generated in the United Kingdom and Ireland through semi-structured qualitative interviews conducted via secure video-conferencing platforms. Participants were invited to freely discuss the ways in which HD affected their daily lives. To ensure cross-cultural relevance, the resulting themes were verified against supplementary qualitative interviews conducted in the Czech Republic (CZ), Germany (GER), Italy (IT), and the Netherlands (NL). Interviews conducted in the United Kingdom (UK) and Ireland (IR) were audio-recorded and transcribed verbatim, with all identifying information removed to preserve participant confidentiality. Data were analysed using theoretical thematic analysis [35], guided by the needs-based model of QoL [25], to identify themes of need fulfilment deficit. Additional methodological details relating to Stage 1 have been described previously [36]. Based on the aforementioned thematic framework, an initial pool of items was developed to reflect the key need-deficit themes identified in the qualitative data.

#### Translation and language adaptation

A dual-panel translation method was employed for the translation of draft items into four languages (Czech, Dutch, German, and Italian). This method is an established way of translating PROMs [37], confirming semantic equivalence and comprehensibility. Each translation of the draft measure required a translation call, conducted with a bilingual representative. The researcher discussed in detail each item and instruction to ensure the representative was familiar with the intended meanings. As the bilingual expert translated each item, providing a back translation, discussions took place to determine whether it was suitably equivalent in meaning, with alternatives given if a direct translation was inappropriate. Once translations, or translation options, were agreed upon by both parties, translations were taken to a lay panel (n = 5 per country) to provide a more objective perspective on the comprehensibility. The lay sample included participants from a variety of professions and a range of ages. Lay interviewees had no diagnosis of HD, as item content relevance was not assessed at this stage. Where interviewees could not decide upon a preferred translation, options would be taken for further testing in the next stage.

#### Stage 2: Assessment of face and content validity

Cognitive debriefing interviews (CDIs) are used to refine measure items and instructions with the input of members of the relevant disease group, using a semi-structured interview assessing acceptability, comprehensibility and item content relevance [38, 39]. The interviewer guided HD patients through the draft measure, noting hesitations or comments. After completion of the measure, the researcher consulted an interview schedule containing questions relating to specific items that surfaced either a.) in lay interviews for countries in which a translation was required or b.) during the item generation process and/or in other CDI interviews. Participants were also encouraged to provide any additional feedback or suggestions for improvement.

#### Stage 3: Validation survey

Eligible participants were sent a survey pack by their local HD centres in the participating sites, with recruitment guided by the local research teams in the respective centres. The survey pack contained a letter of invitation, a participant information sheet, a demographic questionnaire, the draft version of the HD-mQoL and a copy of the Nottingham Health Profile (NHP) [40] as a comparator measure. The NHP is a 38-item measure of distress consisting of six scales: energy, pain, emotional reactions, sleep disturbance, social isolation and physical mobility. Patients who completed and returned the first pack were sent a second pack 2 weeks later, to assess reproducibility. This included the HD-mQoL and a shorter demographic questionnaire.

Participants were further examined using the standardised Unified Huntington’s Disease Rating Scale (UHDRS) assessment protocol [34], which includes neuropsychological, motor, and functional evaluations. All tests and clinical examinations were performed by trained and experienced personnel (e.g., clinicians and psychologists) in the participants’ local language. Motor impairment was assessed using the Total Motor Score (TMS), which evaluates the presence and severity of neurological motor symptoms associated with HD. The TMS ranges from 0 to 124 points, with higher scores indicating more severe motor impairment [41]. Functional status was assessed using three components of the UHDRS [34]: the Total Functional Capacity (TFC) [42], the Functional Assessment Scale (FAS), and the Independence Scale (IS). The TFC consists of five items—occupation, finances, domestic chores, activities of daily living, and level of care—and ranges from 0 to 13 [42]. The FAS includes 25 yes/no items assessing the ability to perform common daily activities (range 0–25). The IS provides a single score reflecting the level of independence, ranging from 10 to 100. For all three measures, lower scores indicate greater functional impairment. The cognitive domain was assessed using the Symbol Digit Modalities Test (SDMT). Biological disease burden was estimated using CAP100, calculated as the product of excess CAG repeat length and age, as described by Warner et al. [43]. The Prognostic Index for HD (PIN) was calculated as a composite measure incorporating age, CAG repeat length, motor signs, and cognitive performance to estimate disease progression risk [44].

### Statistical analysis

Analyses using the data collected from Stage 3 were conducted within two complementary frameworks: first, Rasch Measurement Theory (RMT), to examine the measure’s structural validity and measurement precision, followed by Classical Test Theory (CTT), to assess reliability and construct validity.

#### Determining the final item set: Rasch measurement theory

RMT was selected as the principal analytic framework due to its capacity to construct unidimensional scales with interval-level measurement properties and its provision of detailed item performance diagnostics. This approach enables the identification of a parsimonious set of items that function as a unidimensional measure, demonstrating strong construct validity and robust measurement properties.

RMT provides a rigorous statistical method for transforming ordinal-level questionnaire responses into interval-level measurement, enabling the construction of measures that support objective comparisons across individuals and groups [45, 46]. Within this framework, both respondents and items are positioned along the same latent continuum representing the underlying theoretical construct, in this case QoL. This alignment allows for meaningful interpretation of the patient’s level of the trait relative to the difficulty level of each item.

To validate the HD-mQoL measure, a series of diagnostic tests were applied to assess the extent to which the data conformed to the assumptions of the Rasch model. Each test targeted a specific psychometric property of the measure. The following were assessed in the evaluation process:

1. *Fit statistics*. Overall model fit was assessed using the item–trait interaction chi-square test, evaluating the invariance of item difficulty across different levels of the trait. These tests examined whether the observed item responses across groups of respondents with varying trait levels align with the expected responses under the Rasch model [47]. In addition, standardised item and person residuals were examined to evaluate the discrepancy between observed responses and model expectations. In this case, the chi-square and *F* tests were used to detect misfitting items. Fit residuals within the range ± 2.5 and non-significant *p* values from the chi-square and *F* tests were considered indicative of acceptable data-model fit. Furthermore, item characteristic curves (ICCs) and observed response patterns were inspected to assess the degree of agreement between the model expectations and the data collected [48].
2. *Person-separation index (PSI)*. The PSI was used to evaluate the ability of the measure to discriminate between individuals with varying levels of QoL. The threshold of 0.70 was set as the minimum acceptable value, indicating the measure can reliably distinguish between at least two strata of respondents [49]. This threshold is generally accepted as the minimum level required for group-level interpretation [50].
3. *Local independence*. Local independence checks were conducted by analysing residual correlations. The violation of this assumption suggests that item pairs share some variance beyond what is explained by the underlying latent trait, indicating potential redundancy or multidimensionality. Significant residual correlations were interpreted as evidence of local dependency in this study. A threshold of 0.20 above the average residual correlation across all item pairs was used to flag potential issues with local independence [51]. Item pairs exceeding this threshold required further action, with either one item being removed or both correlated items being grouped into a subset.
4. *Unidimensionality*. To evaluate the assumption of unidimensionality, a paired *t* test procedure was employed. This involves comparing person estimates derived from two subsets of items that exhibit the most divergent loadings obtained from a principal component analysis (PCA) of residuals [52]. If the measure is unidimensional, then individual person estimates calculated from different subsets of items (even from those with the most contrasting loadings) should remain consistent. The first subset contains items with the highest positive loadings, with the other consisting of items with the highest negative loadings on the first residual component. The person estimates on the latent trait are then derived separately for each subset. Paired *t* tests are conducted to compare these estimates for each individual. If fewer than 5% of the *t* tests yield statistically significant differences (p < 0.05), unidimensionality is supported. This threshold is commonly used as the criterion to indicate that any observed differences are likely due to chance rather than a violation of unidimensionality. In addition, a 95% confidence interval for a binomial test of the proportion is calculated based on the observed number of significant *t* tests. It is assumed that the lower bound of this confidence interval should fall below the 5% threshold to further support the assumption of unidimensionality.
5. *Differential item functioning (DIF)*. DIF analysis was performed to assess whether item parameters remained invariant across different groups of patients. In this study, the DIF was assessed in relation to selected respondent characteristics. Items exhibiting significant DIF were flagged as potentially biased, indicating that their inclusion in the measure could affect measure validity across diverse populations [53]. An ANOVA of standardised residuals was carried out to examine DIF by gender (males vs females), age group (below the median age vs the median age and above), country (UK, DE, CZ, IT), living situation of the patient (whether lives with a spouse/partner or other living situation), ethnicity (dominant majority of the given country vs other backgrounds), employment status (employed vs not employed) and care (care provided vs not). An ANOVA *p* value, adjusted using the Bonferroni method, was used to determine the presence of DIF.
6. *Targeting and item locations*. The alignment between item difficulty and person ability was visually inspected using person–item maps. This analysis assessed whether the items covered the full range of the latent trait and whether the item hierarchy matched theoretical expectations.

The analyses conducted within the Rasch modelling framework followed an iterative process aimed at refining the instrument to ensure both statistical and theoretical strength. At each stage of the analysis, items that demonstrated misfit, defined as exhibiting response patterns inconsistent with the Rasch model, were carefully examined. Depending on the nature and extent of the misfit, one of two corrective actions was taken. Either the problematic item was removed from the item pool, or a data transformation was applied to address the issue. Importantly, only one modification was implemented per iteration to isolate the effects of each change. Following each adjustment, the dataset was re-evaluated to verify that the assumptions of the Rasch model were met. This cycle of evaluation and refinement continued until the remaining set of items demonstrated acceptable fit statistics and aligned with theoretical expectations.

All statistical tests were Bonferroni-adjusted to control for the increased risk of Type I error due to multiple comparisons. This conservative correction ensured that the probability of falsely identifying a misfitting item remained within acceptable limits.

#### Classical test theory

*Internal consistency*. The psychometric properties of the measure were further evaluated using CTT. Internal consistency reliability was assessed using Cronbach’s alpha coefficient, which estimates the degree to which items within the measure are inter-related. A high alpha value indicates that the items function cohesively, supporting the reliability of the measure [54].

*Test–retest*. Time stability of the measure was assessed using Spearman’s rank correlation coefficient, calculated from raw scores obtained from the same participants at baseline and follow-up. Participants who reported significant changes in their perceived general health or disease severity between timepoints were excluded from this analysis to ensure the stability of the underlying construct. Given the ordinal nature of the data, Spearman’s rho was chosen as the appropriate non-parametric measure. A correlation coefficient of 0.85 or higher is generally considered acceptable for instruments intended for use in clinical trials or for monitoring individual patients over time [55, 56].

*Convergent validity*. This was evaluated by examining the relationship between scores and the subscales of the NHP. It was hypothesised that moderate to high correlations would be observed between the HD-mQoL and the NHP domains most relevant to HD, for example, impaired mobility and emotional well-being.

*Discriminative (known-groups) validity*. This was assessed by comparing HD-mQoL scores across groups of participants categorised by self-reported disease severity (mild, moderate, severe, very severe) and general health status (very good, good, fair, poor). To test for statistically significant differences between these independent groups, the Kruskal–Wallis one-way analysis of variance for comparisons involving three or more groups was used.

Psychometric testing was performed using the SPSS v29.0 statistical package. Rasch modelling was performed in RUMM2030 software v5.4.

## Results

### Item generation

A total of 30 qualitative interviews (UK n = 25, IE n = 5, aged 33–75, 56.7% male) were conducted to generate item content. A further 21 qualitative interviews took place in the Czech Republic (n = 5), Germany (n = 7), Italy (n = 5) and the Netherlands (n = 4) (aged 36–67, 33.3% male). Summary reports from these supporting interviews provided sufficient evidence that themes were consistent across countries and not culturally bound.

Analyses identified 597 statements reflecting the impact HD has on the lives of participants. A total of 11 themes related to how HD inhibited need fulfilment were established using these statements. Amongst the most frequently discussed were ‘*autonomy’*, *‘safety and security’*, *‘role’*, *‘interests and hobbies*’, and *‘esteem’*. Further details regarding the qualitative analysis results have been published previously [36].

Based on these themes and associated statements, 81 draft items were initially constructed. Items showing conceptual overlap or redundancy were removed after expert review, resulting in 50 draft items representing all major themes, which were taken forward to the next stage.

### Language adaptation

The 50 draft items were translated into Czech, Dutch, German, and Italian in preparation for cross-national testing using a dual-panel translation method. Initial translation panels revealed no major difficulties, though some linguistic nuances required adjustment. For example, the phrase ‘*follow stories on television’* was problematic in the Czech Republic, Germany and the Netherlands: the verb “*follow*” was interpreted too literally in Czech and German, and “stories” translated ambiguously in Dutch. The term “*storyline*” was ultimately adopted as the preferred alternative.

Lay testing (n = 5 per country, total n = 20) confirmed comprehensibility and that item meaning was maintained. For items and instructions in which the language was unclear, alternative translations were offered. One item, *‘I’m limited in the places I can go’,* was removed at this stage, as it was difficult to translate and had significant conceptual overlap with the item *‘I can’t go to the places I want to go’*. A total of 49 draft items remained after this stage.

### Assessment of content and face validity

A total of 62 CDIs took place to assess for face and content validity. The Netherlands was unable to continue participation from this point due to a lack of resources and, therefore, did not complete any CDIs. Across the remaining five countries (Czech Republic, Germany, Ireland, Italy, and the United Kingdom), 62 participants with manifest HD (54.8% male; aged 23–78 years, mean = 49.4) completed CDIs. The majority were unemployed (71.0%), reported *good* health (45.2%), and described their disease severity as *mild* (71.0%). Full sample characteristics by country are presented in the Supplementary Material Table 1.

After all interviews were complete and reports were reviewed, items that were consistently problematic across countries were flagged for removal if items were also identified as statistically misfitting in the next stage. Several items were adapted in response to CDI feedback. In the UK, 1 instruction and 11 items were adapted; in the Czech Republic, 1 instruction and 3 items were changed; in Germany, 1 instruction and 6 items were adapted; and in Italy, 8 items were changed. At this stage, one item was also removed, as it was perceived to be a truism (*I can’t control what is happening to me*), and one was added, as it was noted that this item more accurately reflected statements from the qualitative interviews (*I don’t answer the door to people I’m not expecting)* when compared to an existing item (*I don’t answer the door to anyone)*. While ostensibly both items present with significant overlap in content, redundancy was purposefully built into these two draft items to facilitate the selection of the most appropriately worded item when examined in the next stage. A 49-item draft measure was taken to the final stage.

### Validation survey

The validation survey was completed by 238 participants with manifest HD (59% male; age range 20–83 years; see Table 1 for demographic characteristics). No major demographic differences were identified between participants recruited across the participating countries. Of these, 181 participants (76%) completed the second administration 2 weeks later to assess test–retest reliability. Ireland did not participate in this phase of the study due to a lack of resources.

**Table 1:** Demographic characteristics of manifest HD sample in stage 3 (Validation Survey)

Characteristics	UK n = 74	Czech Republic n = 26	Germany n = 105	Italy n = 33	Total N = 238
**Sex n (%)**					
Female	33 (45.2)	7 (26.9)	43 (41.0)	13 (39.4)	96 (40.5)
Male	40 (54.8)	19 (73.1)	62 (59.1)	20 (60.6)	141 (59.5)
Age median (IQR)	58 (52–66)	50 (43–55)	56 (48–63)	51 (40–59)	55 (48–63)
Living situation n (%)*					
Live alone	9 (12.3)	1 (3.8)	18 (17.3)	1 (3.0)	29 (12.3)
Spouse partner	58 (79.5)	13 (50.0)	72 (69.2)	23 (69.7)	166 (70.3)
Children under 18	7 (9.6)	1 (3.8)	12 (11.5)	2 (6.1)	22 (9.3)
Children over 18	8 (11.0)	5 (19.2)	18 (17.3)	5 (15.2)	36 (15.3)
Parents	5 (6.8)	7 (26.9)	8 (7.7)	6 (18.2)	26 (11.0)
Other	1 (1.4)	4 (15.4)	7 (6.7)	3 (9.1)	15 (6.4)
Prefer not to say	0 (0.0)	2 (7.7)	0 (0.0)	0 (0.0)	2 (0.8)
**Employment n (%)**					
Full time	8 (11.0)	1 (3.8)	9 (8.7)	15 (45.5)	33 (14.0)
Part time	2 (2.7)	2 (7.7)	11 (10.6)	3 (9.1)	18 (7.6)
Self-employed	0 (0.0)	0 (0.0)	1 (1.0)	1 (3.0)	2 (0.8)
Not employed	63 (86.3)	23 (88.5)	84 (80.8)	14 (42.4)	184 (78.0)
**Care provided n (%)**					
No	20 (27.4)	4 (15.4)	13 (12.6)	20 (60.6)	57 (24.3)
Yes	53 (72.6)	22 (84.6)	90 (87.4)	13 (39.4)	178 (75.7)
Care provider n (%) *					
Other family member	15 (20.5)	8 (30.8)	37 (35.9)	0 (0)	60 (25.5)
Spouse/partner	43 (58.9)	11 (42.3)	65 (63.1)	13 (39.4)	132 (56.2)
Professional carer	3 (4.1)	7 (26.9)	19 (18.4)	0 (0)	29 (12.3)
Other	2 (2.7)	0 (0)	8 (7.8)	0 (0)	10 (4.3)
Not applicable	20 (27.4)	4 (15.4)	13 (12.6)	20 (60.6)	57 (24.3)
**Disease severity n (%)**					
Mild	29 (40.3)	8 (30.8)	37 (36.3)	24 (75.0)	98 (42.2)
Moderate	35 (48.6)	13 (50.0)	33 (32.4)	8 (25.0)	89 (38.4)
Severe	8 (11.1)	5 (19.2)	25 (24.5)	0 (0.0)	38 (16.4)
Very severe	0 (0.0)	0 (0.0)	7 (6.9)	0 (0.0)	7 (3.0)
**General health n (%)**					
Poor	10 (13.9)	2 (7.7)	19 (18.1)	2 (6.1)	33 (14.0)
Fair	22 (30.6)	12 (46.2)	27 (25.7)	11 (33.3)	72 (30.5)
Good	33 (45.8)	10 (38.5)	44 (41.9)	12 (36.4)	99 (41.9)
Very good	7 (9.7)	2 (7.7)	15 (14.3)	8 (24.2)	32 (13.6)

*indicates that for these variables’ participants were able to tick multiple answers. *mHD* manifest Huntington´s Disease, *N* number of participants, *IQR* interquartile range

The study cohort displayed clinical characteristics typical of manifest HD, with a median CAG repeat length of 43 (interquartile range IQR 42–45). Motor impairment was moderate, with a median UHDRS–TMS of 30 [21–41]. Functional impairment was evident, reflected by a median TFC score of 9 [7–12], a median FAS score of 20 [16–24], and a median IS of 80 (70–90). Cognitive processing speed was reduced, as indicated by a median SDMT score of 23 [17–31]. Disease burden was substantial, with CAP100 values showing a median of 110.02 (IQR 99.61–120.26) and a PIN score with a median of 3.02 (2.17–3.81), consistent with a cohort predominantly composed of individuals with manifest HD. Detailed clinical characteristics are provided in Supplementary Material Table 2.

**Table 2:** Summary of overall Rasch model fit statistics for the final 23-item HD-mQoL measure

	Item-trait interaction chi-square test	Person-separation index (PSI)	Item-fit residuals		Person-fit residuals		Unidimensionality *t* tests: proportion of significant paired *t* tests
			Mean	SD	Mean	SD	
HD-mQoL measure	p = 0.391	0.853	0.405	0.982	-0.180	0.828	0.017
Target values	> 0.05	> 0.70	< 0.50	< 1.40	< 0.50	< 1.40	< 0.05

### Results of statistical analyses.

The application of RMT to the initial pool of 49 candidate items resulted in the removal of 26 items to establish the final version of the HD-mQoL.

#### Item reduction process.

The Rasch analysis revealed that seven items violated assumptions of the theoretical model due to having item fit residuals outside the range ± 2.5, and significant *p* values in the statistical tests applied. Thirteen items were removed because of high residual correlations with other items, thus violating the assumption of local independence. Four items demonstrated significant DIF and were removed. Three items demonstrated more than one type of issue, such as misfit, local dependence or DIF. Finally, four items were excluded from the measure, because they were found to be redundant, offering overlapping information with other items. A review of the remaining items confirmed that all essential content areas were still adequately represented, preserving face and content validity.

#### Final data-model fit.

**Fig. 2:**
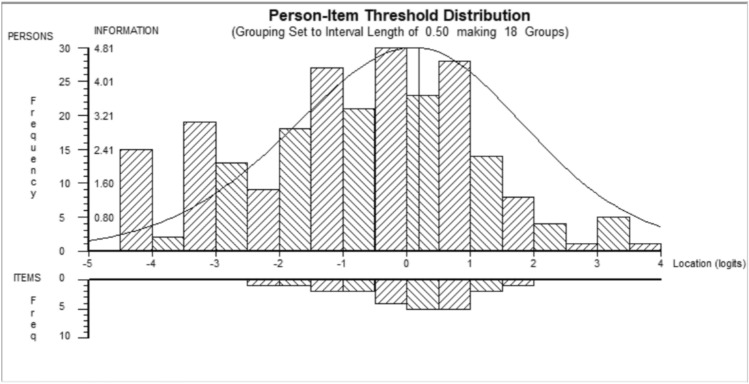
Item-person map for HD-mQoL measure. The top section of the figure presents the distribution of patients on the interval measure with logit units. The bottom section shows the range of item locations

The overall Rasch fit statistics for the final 23-item measure are shown in Table 2. These confirm that the measure fits the Rasch model and provides robust evidence of unidimensionality. Figure 2 shows that the items covered a wide range of severities and were well-matched to the severity level of the sample. Applied tests based on the results of PCA and paired *t* tests confirmed unidimensionality of the proposed measure. High scores on the HD-mQoL measure represent a more severe impact of HD on the QoL of patients. Could you please insert Table 2 above here, at the moment it is in wrong section.

Classical psychometric analyses.

The final set of 23 items demonstrated excellent reliability. Internal consistency, as assessed by Cronbach’s alpha, was exceptionally high reaching 0.91 at Time 1 and 0.92 at Time 2. These values indicate a strong degree of inter-relatedness amongst the items, suggesting that they consistently measure QoL across both timepoints. Test–retest reliability measured by the Spearman correlation coefficient was also high (0.871, p < 0.001; N = 156). Such a high reliability coefficient supports the measure’s stability over time.

Table 3 shows descriptive statistics for the measures used in the study. Some floor effects were found in the HD-mQoL measure (7% of patients scored the minimum possible score), with the number of patients scoring the maximum possible score also being very small (< 1%). In contrast, significant floor effects (36.5%–73.4%) were found for the NHP, showing the limitations of applying generic PROMs to rare diseases, such as HD.

**Table 3:** Descriptive and reliability statistics for the HD-mQoL and comparator measure NHP

	N	Median (IQR)	Range	% scoring minimum	% scoring maximum
HD-mQoL measure	215	8 (3–14)	0–23	7.0	0.5
**NHP subscales**					
Energy	224	33 (0–67)	0–100	39.7	17.4
Pain	222	0 (0–13)	0–100	73.4	1.4
Emotional reactions	217	11 (0–33)	0–100	37.3	1.4
Sleep	224	20 (0–40)	0–100	42.4	4.0
Social isolation	216	20 (0–40)	0–100	48.6	1.4
Physical mobility	219	13 (0–50)	0–100	36.5	0.9

Huntington’s Disease Manifest Quality of Life (HD-mQoL) measure, Nottingham Health Profile (NHP) [40].

Table 4 shows the correlations between HD-mQoL scores and those on the NHP sections, across both physical and emotional impairments. All correlations with the HD-mQoL are positive with a moderate to high magnitude, suggesting that all these NHP domains influence the QoL of HD patients to some extent. However, stronger correlations were observed with the NHP domains of energy, emotional reactions, physical mobility and social isolation, whereas weaker levels of association were found with the pain and sleep scales. These results align with the typical patterns observed in people with HD, known to impact social isolation, mobility, emotional well-being and energy [57, 58].

**Table 4:** Spearman rank correlation coefficients were calculated between scores on HD-mQoL (time1) and NHP sections

	Correlation with HD-mQoL	p	N
**NHP subscale**			
Energy	0.669	< 0.001	206
Pain	0.328	< 0.001	203
Emotional reactions	0.670	< 0.001	199
Sleep	0.327	< 0.001	204
Social isolation	0.724	< 0.001	199
Physical mobility	0.697	< 0.001	201

All correlations are positive and statistically significant, supporting the convergent validity of the HD-mQoL. Huntington’s Disease Manifest Quality of Life (HD-mQoL) measure, Nottingham Health Profile (NHP) (40).

**Fig. 3:**
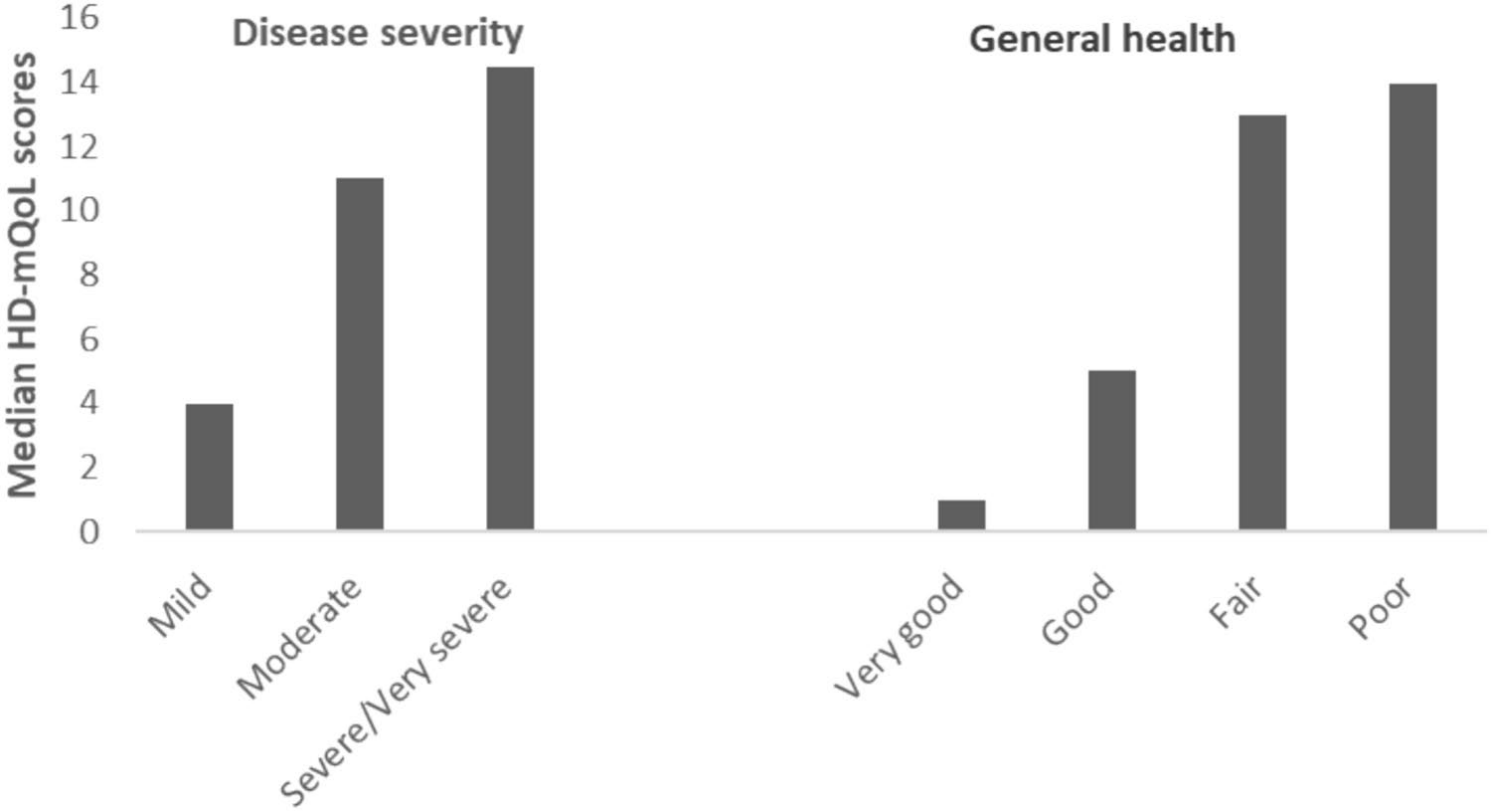
Median HD-mQoL scores by disease severity and self-reported general health

Significant differences in HD-mQoL scores were observed between patients grouped by disease severity and general health (p < 0.001) (see Fig. 3). This demonstrates that the HD-mQoL was able to distinguish successfully between the groups of known importance. Following the Kruskal–Wallis tests, pairwise Dunn’s post hoc comparisons were applied and confirmed statistically significant differences between all groups. Specifically, the comparisons revealed significant differences between the ‘mild’ and ‘moderate’ groups (Bonferroni-adjusted p < 0.001), between ‘mild’ and ‘severe/very severe’ (p < 0.001), and between ‘moderate’ and ‘severe/very severe’ (p = 0.024). Similar findings were observed in the post hoc comparisons based on categories defined by the general health question. The results indicated statistically significant differences (p < 0.010) across all pairs, with the exception of the ‘fair’ vs ‘poor’ comparison, which did not reach significance (p = 1.000).

The final measure included items across a range of logit locations, with the content covering a variety of underlying needs (see Table 5).

**Table 5:** Examples of final HD-mQoL items with corresponding underlying needs and Rasch item locations

Item	Underlying Need	Location (logit)
I’m unable to do things spontaneously	Autonomy	-0.526
I neglect my friends	Socialising	0.278
I’m unable to do my hobbies	Pleasure	-0.428
I feel vulnerable when I’m on my own	Safety and Security	0.467

## Discussion

PROMs are essential for evaluating the impact of disease and treatment from the patient’s perspective, yet traditional symptom- and function-based instruments may not fully capture the complex, lived experience of individuals with HD, where emotional, cognitive, and social factors interact dynamically with physical decline [9]. Due to factors such as anosognosia and wide variability in how patients perceive the importance of specific symptoms, relying solely on functional measures risks misrepresenting the patient’s QoL [11]. As clinical trials, aimed at slowing HD progression, continue to advance, there is a growing need for a sensitive, patient-centric tool capable of detecting subtle yet clinically meaningful changes in QoL [59]. The present study addresses this gap through the development and validation of the first needs-based, disease-specific PROM designed specifically for individuals with manifest HD.

The HD-mQoL, a concise, 23-item, unidimensional measure of QoL, captures the extent to which fundamental human needs are met or unmet as a consequence of HD. The items were derived directly from patient interviews and refined through cross-national cognitive debriefing, ensuring strong conceptual relevance and patient-centredness. The structure and scaling properties of the HD-mQoL are supported by the application of Rasch analysis, thereby confirming unidimensionality and enabling interval-level measurement of QoL. The Rasch framework enabled a detailed evaluation of item performance, allowing for the removal of 26 draft items through an iterative process that combined statistical evidence with qualitative feedback. This integrated approach ensured that the final scale retained both psychometric robustness and conceptual relevance to the patient experience. This feature is particularly valuable for clinical and research applications, allowing for meaningful comparisons across patients, timepoints, and interventions [60]. The utilisation of dichotomous response options has also been demonstrated to enhance the ease of completion for patients with cognitive challenges and facilitate straightforward scoring and interpretation by clinicians [61].

In addition, this work was strengthened by the collaborative, international development process. Through CDIs conducted in the Czech Republic, Germany and Italy, the relevance and clarity of item content were confirmed across cultural contexts. Crucially, Rasch analysis demonstrated no evidence of DIF by country in the final measure, supporting the HD-mQoL’s cross-cultural validity and its potential for use in future multinational trials. Furthermore, the successful translation process demonstrated that future linguistic adaptations can be readily implemented using established dual-panel translation procedures.

The HD-mQoL is, therefore, recommended as a robust measure for assessing QoL in patients with manifest HD. It is considered suitable for use in both clinical practice and research settings to monitor QoL changes over time.

## Limitations

First, during the postal survey phase, some missing data were observed. This was likely due to suboptimal placement of instructions and layout, as well as the length of the questionnaire, which may have caused confusion for participants with cognitive impairments or motor difficulties. Similar issues have been reported in the development of other HD-specific QoL instruments, such as the H-QoL-I [17], emphasising the need to consider cognitive processing limitations at all stages of PROM development. To address this, the final version of the HD-mQoL incorporates clearer instructions, simplified formatting, and a substantially reduced item count following the removal of 26 items. These revisions are expected to enhance usability and completion accuracy, particularly among individuals with cognitive or fine motor challenges.

Second, although the HD-mQoL reflects the experiences of the largest HD subgroup [1], patients with mild to moderate manifest disease, it was challenging to include individuals with severe HD during item generation. Recruiting and interviewing those with advanced disease presents substantial ethical and practical challenges, including difficulties with informed consent and communication [62]. While proxy interviews could have been employed, prior evidence suggests that proxies tend to underestimate patients’ QoL [63, 64], which could have compromised the patient-centred nature of the measure. This complex methodological issue warrants further discussion within the HD research community and reflects the broader challenge of incorporating the voices of individuals with severe cognitive and psychiatric impairments in research. While the generalisability of the findings to this population is, therefore, reduced, the current study did, however, integrate the development of a proxy version of the HD-mQoL into the HEALTHE-RND project [65]; the patient-derived item content but is designed to be completed by a proxy. This measure will provide a reasonable reflection of patient-centric QoL for patients with advanced HD unable to complete the questionnaire themselves.

An additional limitation regarding the study inclusion criteria should be acknowledged. At the conception of the study, the most widely accepted clinical definition of manifest HD was a DCL of 4. However, when the study commenced in 2020, the HD-ISS staging system [66] had not yet been fully developed and, therefore, could not be incorporated into the study design. While the clinical characteristics presented in Supplementary Table 2 indicate how the sample aligns with the HD-ISS stages, the use of HD-ISS staging during recruitment would have been preferable. Furthermore, a DCL of 4 excludes individuals experiencing cognitive symptoms in the absence of motor manifestations. It is hoped that the needs-based QoL measure currently under development for the premanifest population will capture both asymptomatic individuals and those in the early stages of HD presenting with cognitive and/or psychiatric symptoms only.

## Conclusion

The HD-mQoL is a psychometrically sound, patient-centred measure of QoL for individuals with manifest HD. Its needs-based content ensures aspects of QoL beyond symptoms and functioning are captured, offering a meaningful unidimensional outcome metric for a notoriously complex, multi-faceted condition. With demonstrated cross-cultural validity, and applicability for clinical and research use, the HD-mQoL represents a significant advancement in outcome measurement for HD. The HD-mQoL thus provides clinicians and researchers with a reliable, easy-to-use tool for assessing QoL from the patient’s perspective in manifest HD.

## Data Availability

The data that support the findings of this study are available from the corresponding author on reasonable request. The data are not publicly available due to privacy or ethical restrictions.
